# The Portuguese Model of Home Respiratory Care: Healthcare Professionals’ Perspective

**DOI:** 10.3390/healthcare9111523

**Published:** 2021-11-09

**Authors:** Cátia Caneiras, Cristina Jácome, Daniela Oliveira, Emília Moreira, Cláudia Camila Dias, Liliane Mendonça, Sagrario Mayoralas-Alises, João Almeida Fonseca, Salvador Diaz-Lobato, Joan Escarrabill, João Carlos Winck

**Affiliations:** 1 Microbiology Research Laboratory on Environmental Health (EnviHealthMicro Lab), Institute of Environmental Health (ISAMB), Faculty of Medicine, University of Lisbon, 1649-028 Lisbon, Portugal; 2 Institute for Preventive Medicine and Public Health, Faculty of Medicine, University of Lisbon, 1649-028 Lisbon, Portugal; 3 Healthcare Department, Nippon Gases Portugal, 2600-242 Vila Franca de Xira, Portugal; 4 Center for Health Technology and Services Research (CINTESIS), Faculty of Medicine, University of Porto, 4200-450 Porto, Portugal; cjacome@med.up.pt (C.J.); u015011@chsj.min-saude.pt (D.O.); emiliamoreira@med.up.pt (E.M.); ccamiladias@gmail.com (C.C.D.); liliane.mendonca@gmail.com (L.M.); fonseca.ja@gmail.com (J.A.F.); 5 Department of Community Medicine, Information and Health Decision Sciences (MEDCIDS), Faculty of Medicine, University of Porto, 4200-450 Porto, Portugal; 6 Department of Medicine, Faculty of Medicine, University of Porto, 4200-319 Porto, Portugal; jcwinck@mail.telepac.pt; 7 Rheumatology Department, Centro Hospitalar Universitário São João (CHUSJ), 4200-319 Porto, Portugal; 8 Healthcare Management Department, Hospital Quirón Salud San José, 28002 Madrid, Spain; sarimayoralas@gmail.com; 9 Allergy Unit, Instituto and Hospital CUF Porto, 4460-188 Matosinhos, Portugal; 10 Healthcare Department, Nippon Gases Spain, 28020 Madrid, Spain; sdiazlobato@gmail.com; 11 Service of Pneumology, Hospital Universitario Moncloa, 28008 Madrid, Spain; 12 Programa de Atención a la Cronicidad, Hospital Clínic de Barcelona, 08036 Barcelona, Spain; escarrabill@clinic.cat; 13 Master Plan for Respiratory Diseases (Ministry of Health) & Observatory of Home Respiratory Therapies (FORES), 08028 Barcelona, Spain; 14 REDISSEC Health Services Research on Chronic Patients Network, Instituto de Salud Carlos III, 28029 Madrid, Spain

**Keywords:** oxygen inhalation therapy, mechanical ventilation, focus groups, long-term care, quality of health care, Home care services

## Abstract

Patients’ and carers’ views regarding the Portuguese model of home respiratory care were recently described, yet the complementary perspective of healthcare professionals (HCPs) is still to be investigated. Thus, this study explored HCPs experience in the management of patients needing home respiratory therapies (HRT), and their perspective about the Portuguese model. A phenomenological descriptive study, using focus groups, was carried out with 28 HCPs (median 42 y, 68% female) with distinct backgrounds (57% pulmonologists, 29% clinical physiologists, 7% physiotherapists, 7% nurses). Three focus groups were conducted in three regions of Portugal. Thematic analysis was performed by two independent researchers. HCPs have in general a positive view about the organization of the Portuguese model of home respiratory care, which was revealed in four major topics: Prescription (number of references, *n* = 171), Implementation and maintenance (*n* = 162), Carer involvement (*n* = 65) and Quality of healthcare (*n* = 247). Improvements needed were related to patients’ late referral, HRT prescription (usability of the medical electronic prescription system and renewals burden), patients’ education, access to hospital care team, lack of multidisciplinary work and articulation between hospital, primary and home care teams. This study describes the perspective of HCPs about the Portuguese model of home respiratory care and identifies specific points where improvements and reflections are needed. This knowledge may be useful to decision makers improve the current healthcare model.

## 1. Introduction

Home respiratory therapies (HRT), such as long-term oxygen therapy and home mechanical ventilation, are prescribed to improve the health-related quality of life of patients with respiratory disorders such as chronic obstructive pulmonary disease (COPD), pulmonary fibrosis, cystic fibrosis, bronchiectasis, neuromuscular diseases, among others. HRT are one of the most important home healthcare services [1] and patients needing HRT are increasing [2]. This adds to HRT high complexity, characterized by the chronicity of the patient condition itself, the need of patients’ and carers’ training and the involvement of variety of healthcare professionals [3,4]. This scenario poses significant challenges to the capacity of health care services worldwide.

Quality of care is “the extent to which health care services provided to individuals and patient populations improve desired health outcomes. In order to achieve this, health care must be safe, effective, timely, efficient, equitable and people-centered” [5]. A necessary step in the process of maintaining and improving quality is to monitor and evaluate the quality of healthcare in routine clinical practice [2].

A large heterogeneity exists among HRT models in place. Questionnaires and databases from HRT registries or health services were previously used to describe home respiratory care models, although qualitative studies have also been conducted [2,3]. Of relevance, in Europe there is no single home respiratory care model, but instead distinct organizations and delivering approaches exist across countries and even within the same country [2,6]. This creates substantial challenges in using evidence gathered from different contexts to contribute for improvements of the models in place. Thus, studies for each individual model are crucial.

At the Portuguese national health service, an innovative patient-centered home respiratory care model exists to maintain access to HRT, sustainability, and quality of care [7]. The Portuguese model is based on the following pillars: prior approval of providers at national level; geographic equity due the same therapies are available throughout the continental territory; single price by therapy among all providers and competition for the quality of the service provided. There is free choice of provider by the patient [7,8]. Innovatively, a total of 19 home respiratory therapies grouped into (i) home mechanical ventilation (non-invasive ventilation to treat respiratory failure and complex sleep apnea), (ii) oxygen therapy (liquid oxygen, cylinders, conventional concentrator, and portable oxygen concentrators), (iii) aerosol therapy and (iv) other respiratory therapies, as mechanical insufflation-exsufflation (MI-E) are available through specific medical electronic prescription [8]. Considering that this model is in place since 2014, its ongoing assessment is extremely useful to contribute to continuous improvement of the quality of the provided home healthcare service [2].

Quantitative studies have been conducted, which are already informative by pointing out points for improvement, namely the creation of national protocols to standardize procedures (as clinical reports) and of audit practices [6,8]. Yet, qualitative approaches are also of major relevance to explore the perspective of distinct stakeholders–patients, carers and healthcare professionals, regarding the difficulties with implementation of HRT in the real-world setting.

A recent study about the experience of patients receiving HRT and their carers showed a general good perception of the care received, while identifying specific points where improvements are needed: particularly regarding navigability issues, prescriptions renewals burden and articulation between hospital, primary care, and home care teams [9]. However, the perspective of healthcare professionals is yet to be explored.

Therefore, the aim of this study was to explore healthcare professionals’ experience in the management of patients needing HRT, while assessing their perspective about the Portuguese model of home respiratory care.

## 2. Materials and Methods

### 2.1. Study Design

A phenomenological descriptive study [10], using focus groups, was carried out to describe healthcare professionals’ experiences in the management of patients receiving HRT. Focus groups was the selected method due to its ability to enhance interaction amongst participants and to generate a rich understanding of people’s experiences and beliefs [11]. Three focus groups were conducted at three regions of Portugal (North-Porto, Center-Coimbra and South-Lisboa), to gather a national perspective. The study is reported following the Consolidated Criteria for Reporting Qualitative Research (COREQ) [12] and the standards for reporting qualitative research (SRQR) [13].

### 2.2. Ethical Considerations

This study was conducted in line with the Declaration of Helsinki and its protocol received approval from the Ethic Committee of the Unidade Investigação em Ciências da Saúde: Enfermagem from the Escola Superior de Enfermagem de Coimbra (P630-11/2019). All participants gave their written informed consent before any data collection.

### 2.3. Participants

Healthcare professionals were eligible if they were directly involved in the diagnosis and/or management of patients with chronic respiratory failure receiving HRT. There were no exclusion criteria. The research team planned to recruit a convenience sample of 8–10 HCP for each focus group, trying to balance distinct clinical backgrounds. Healthcare professionals were contacted by telephone by a researcher, who informed about the study and invited them to participate in the focus group meeting. A total of 28 healthcare professionals participated in the three focus groups (Table 1). Healthcare professionals (HCPs) were mostly female (68%), with a median age of 42 (percentile 25–percentile 75 37−53) years. More than half were pulmonologists (57%) and 29% clinical physiologists. The majority were working in secondary care settings (79%).

### 2.4. Data Collection

All data collection took place at the three focus group meetings. The focus group meetings were held in the community (hotel conference rooms), outside participants’ healthcare institutions, so HCPs could more freely express their perspective. As this study was explorative in nature, data saturation was not pursued. Before starting the focus groups, a clear explanation on the aim of the study was provided to all participants and consent forms were obtained. Participants completed a brief questionnaire about sociodemographic (gender, age) data and about their clinical background (profession, years of working experience, working sector and setting).

One moderator (LM) conducted all focus groups. LM is a female trained psychologist with a Master in Evidence and Decision in Health. At least two group assistants (CJ, CCD or CC) were present in each focus group to take observational notes of the group interaction and of the most important messages and ideas for analysis. One additional person was present, being responsible for the audio and image recordings. Both moderator and group assistants were experienced in conducting focus groups. Before the start of the study, there was no relationship established between moderator and participants. Before the interview, the moderator, the group assistants and participants had the opportunity to present themselves to create a comfortable atmosphere and “breaking the ice”. Then, focus groups were conducted in a nondirective manner according to a semi-structured discussion guide (Appendix A), with participants gathered in a round table. On average, the focus groups lasted 73 min (range 61–89). Data from the audio recorders was saved to a computer with restricted access only to the researchers and the original audios were deleted from the devices. The focus group were transcribed by one researcher (LM) and checked for accuracy by two other members of the team (CJ and CCD). During transcription, participants’ identification was coded to preserve anonymity.

### 2.5. Data Analysis

Two independent researchers (DO and EM) read the full transcriptions to obtain an overview of the collected data and then performed a thematic qualitative analysis based on the six steps proposed by Braun and Clarke (1 familiarization with the data, 2 generating initial codes, 3 searching for themes, 4 reviewing themes, 5 defining and naming themes and 6 producing the report) [14]. The NVivo 12 plus (QSR International, Melbourne, Australia) software was used. To ensure the reflexivity, the researchers held regular group meetings to reflect and discuss issues related to the study [15]. About one month, after the last focus group meeting, the preliminary focus group results were discussed with 5 healthcare professionals (2 pulmonologists, 2 clinical physiologists, 1 nurse) to ensure credibility and trustworthiness of findings [16]. After this validation procedure, the researchers had a last meeting to reflect on the main issues brough by this study.

To determine the consistency of the qualitative analysis carried out by the two researchers, an inter-rater agreement analysis using percentages of agreement (number of units of agreement divided by the total units of measure within the data item, displayed as a percentage) and Cohen’s kappa (statistical measure which considers the amount of agreement that could be expected to occur through chance) was carried out. One focus group was selected randomly to perform this analysis [17]. The value of Cohen’s k ranges from 0 to 1 and can be categorized as slight (0.0–0.20), fair (0.21–0.40), moderate (0.41–0.60), considerable (0.61–0.80) or almost perfect (≥0.81) agreement [18]. All statistical analyses were carried out using SPSS Statistics (version 26.0; SPSS Inc., Armonk, NY, USA).

## 3. Results

As can be seen from Table 2, four major topics emerged during the analysis of the focus groups. Inter-rater agreement between the two researchers in these major topics was found to be high (percentage of agreement 95–100%, kappa 0.989).

A brief description of each topic and sub-topic is provided below:

### 3.1. Prescription

This topic deals with the experience of HCPs in the prescription of HRT regarding context, roles, administrative issues, and communication with the patient.

#### 3.1.1. Setting and HCP Roles

The prescription and introduction to HRT is mainly made at secondary or tertiary care, commonly by a pulmonologist during a scheduled consultation or a hospitalization, however other physicians, from internal medicine, cardiology, or other medical specialties, can also prescribe (or refer to pulmonology) in the context of a follow up of other health conditions, of an emergency department visit or hospitalization. Patients can be also referred by their general practitioner to secondary care, although this pathway is less common. There is however a general perception of patients’ late referral, which was identified as an issue to be improved. Prescription is based on an electronic prescription system, being only available in the public national health service, which was stressed as a great disadvantage for patients followed up in private hospitals.

*“Referral should occur as soon as possible, which is rarely the case”*

(Male, pulmonologist, 63 y)

*“(…) for patients managed in private hospitals, we cannot prescribe HRT. This is a huge incongruity as patients need to have an appointment in a public hospital to have access to a prescription”*

(Male, pulmonologist, 60 y)

*“(…) the objective of the Medical Electronic Prescription for Home Respiratory Care is the dematerialization of the prescription process”*

(Male, pulmonologist, 60 y)

#### 3.1.2. Education

HCPs inform patients about the HRT being prescribed, its goals and expected benefits. They also inform patients that they are free to choose the home care provider but recognized that commonly this is a “guided choice” as patients rely on HCP’s suggestions to decide. HCPs highlighted that this happens because homecare providers are not involved at this stage and thus patients most of the times do not have any previous knowledge to make an informed decision. When guiding the patient in their choice, HCPs value the company responsiveness in a short time, capacity to anticipate and solve problems, the quality of the feedback provided, the ability to manage patients with complex disease, and the devices and interfaces used.

*“we recognize what is really impacting patients’ daily life, if it is dyspnea, fatigue, headaches, and we try to explain the benefits of the HRT using those symptoms”*

(Female, pulmonologist, 35 y)

*“the patient is the one that decides the homecare provider, but in case of doubt, the physician is the best positioned to make a suggestion”*

(Male, pulmonologist, 63 y)

*“(…) those homecare providers that offer the best follow up, the best monitoring, are the ones we will recommend”*

(Female, pulmonologist, 35 y)

They also inform the patient of the signs/symptoms of an exacerbation and actions to be taken in this situation. Safety information related with HRT and of possible adverse events are not usually provided at this phase. Nevertheless, HCPs reported that content of the information provided in this first contact differs based on patients’ literacy, which is generally low, due to time constraints. It is clear from HCPs reports that structured education interventions are not in place. They reported that during consultations there is space for doubts, and common questions are related with the lifelong need of treatment and about severity of the disease as patients link the need of HRT to end-of-life stages. The written information provided is the one present in the prescription forms (type of HRT, when to use, how many hours). HCP referred initiatives to promote the sharing of experiences between patients were not common, that patients learn from each other in the waiting room mainly. They referred the importance of patients’ associations (Portuguese Association of People with COPD and Other Chronic Respiratory Diseases-RESPIRA, Portuguese Association of Neuromuscular Diseases, Portuguese Association of Amyotrophic Sclerosis) in disseminating useful information.

*“the basic information, how many hours use, when to use, is in the prescription form delivered in paper to the patient”*

(Male, pulmonologist, 39 y)

*“we lose a lot of time with these patients [with low literacy]”*

(Female, pulmonologist, 53 y)

*“Patients learn in the waiting room, because they find other patients using HRT and ask them “how do you do when…?”*

(Male, pulmonologist, 63 y)

### 3.2. Implementation and Maintenance

This topic deals with the experience of HCPs in the initial implementation of HRT, specifically the possible settings, each HCP role and education at this time point, but also their experience with factors related to patients’ maintenance of the therapies, namely adherence, quality of life impact and carer involvement.

#### 3.2.1. Setting and HCP Roles

The healthcare team involved in the implementation and maintenance varies depending on the setting and region. The hospital core team are, in general, pulmonologists, nurses and clinical physiologists. Other allied health professionals, such as physiotherapists, nutritionists, psychologists, social assistants may also be involved, but this is not the standardized approach. Introduction to HRT occurs preferentially at hospital, during hospitalizations, dedicated appointments, or trials at respiratory labs, in which nurses and clinical physiologists are involved in adapting the patient to the therapy and for reinforcing education. But many times this is not possible due to staff constraints and the first contact with the prescribed HRT is at home with HCPs of the homecare provider (clinical physiologist, physiotherapist mainly).

*“(…) adaptation to ventilation or oxygen therapy is made at the hospital”*

(Female, clinical physiologist, 37 y)

*“(…) we don’t have enough technical staff to make adaptation to the therapy at the hospital, so the patient received a prescribed and the homecare provider perform the adaptation”*

(Female, pulmonologist, 39 y)

Irrespective of the initial pathway, is at the first home visit that the adjustment of the HRT is made, with HCP’s demonstrating the respiratory expertise technique, exploring the best locations for the equipment (safety) and testing different interfaces in order to better personalize the interface adjustment and latter promote the adherence. After this adaptation (one week), a follow up phone contact is made by the HCP’s of home provider to evaluate patients’ adaptation to the HRT and clarify doubts, and if need a home visit is scheduled.

*“(…) is indeed in patient’s home, in his room, that therapy and equipment needs to be personally adjusted (…)”*

(Male, pulmonologist, 63 y)

*“(…) sometimes what is achieved at the lab is not reproducible at home, enhancing the relevance of the home care team”*

(Female, clinical physiologist, 39 y)

*“(…) we demonstrate the device, we test which interface is best suited to the patient and, more importantly, we talk about the therapy”*

(Male, Clinical physiologist, 29 y)

*“After one week, I call to the patient and ask how therapy is going, and whenever he has doubts or complaints, I immediately schedule a home visit”*

(Female, clinical physiologist, 24 y)

#### 3.2.2. Education

At this stage, the benefits of the HRT are again explained and reinforced by the homecare teams, together with the importance of correctly adhering to obtain the expected benefit. Patients and carers are informed about the home care team support and contacts (available 24/7), equipment handling, namely safety issues and cleaning practices, and how to act in normal or critical situations (e.g., back up equipment, oxygen replacement). HCPs recognize that a lot of information is provided at this stage and that it needs to be constantly reinforced both verbally and through written information (patient manual) at the different settings (e.g., phone contacts, home visits, appointments at the hospital).

*“a huge amount of information is provided at the beginning of the therapy”*

(Female, clinical physiologist, 29 y)

*“at home, we deliver a manual with the therapy information and brief device instruction that describe all safety issues (…)”*

(Male, clinical physiologist, 38 y)

*“(…) in the next hospital visit we will reinforce, safety issues, such as back up ventilator, ambu use, basic life support”*

(Male, physiotherapist, 40 y)

#### 3.2.3. Adherence

HCPs acknowledge that distinct factors contribute to different adherence behaviors. Patients with more severe symptoms, experiencing benefits of HRT during a hospitalization, or those with easily adaptation to the equipment are in general more adherent. Young patients have more difficult to perform the therapy all the prescribed hours as they are commonly more active (employment, recreation, etc.) than older patients and also feel more embarrassed about using the consumables associated with oxygen and ventilation (face and nasal masks or nasal cannulas).

*“(…) the more severe patients are the ones better adapting (…)”*

(Female, pulmonologist, 43 y)

*“(…) patients that already felt the benefits of ventilation during a hospitalization, adapt better”*

(Male, pulmonologist, 60 y)

*“the youngest patients feel restricted of having to perform the therapy those daily hours”*

(Male, pulmonologist, 39 y)

#### 3.2.4. Quality of Life Impact

HCP reported the benefits of the HRT in patients’ quality of life, namely the improved physical and respiratory condition to perform activities of daily living. But they also focused on the negative impact, namely the social stigma associated with HRT, the travel restrictions, particularly air travels; and the emotional impact (anxiety, depression, fears, concerns related with future unpredictability). Along the focus group, the HCPs made suggestions to improve society awareness about HRT emerged.

*“These therapies cause an important psychological impact in the patient, in his quality of life”*

(Male pulmonologist, 65 y)

*“the enormous heterogeneity among airlines related with long-term oxygen therapy, with different policies and costs, makes tremendously difficult to schedule a travel”*

(Male, pulmonologist, 60 y)

*“patients have a lot of fears and insecurities”*

(Female, nurse, 47 y)

*“(…) there is a general low awareness of the society regarding HRT. We see campaigns about asthma, about COPD, but I never saw a campaign about LTOT or home ventilation”*

(Male, pulmonologist, 39 y)

### 3.3. Carer Involvement

This topic is related with HCP view about the integration of carers in the care-pathway. Carer involvement was perceived as positive in the various stages of the HRT: prescription, implementation, and maintenance, not only because they provided physical and emotional support to the patient, but also because they facilitate the communication with the healthcare team and participate in the patients’ education. Nevertheless, different views coexisted: some considered the involvement of the carer dependent on the patient’s autonomy, while others consider it essential irrespective of it. HCPs also referred that sometimes carers’ excessive support and protection jeopardizes patients’ independence and autonomy.

*“carers are involved in the talk, when information is provided”*

(Female, pulmonologist, 41 y)

*“through the carer we try to understand the difficulties in the day-to-day management of the therapy”*

(Female, pulmonologist, 41 y)

*“Carers’ excessive support may be negative as it prevents patients’ autonomy/independence”*

(Male, pulmonologist, 63 y)

### 3.4. Quality of the Healthcare

This topic describes HCPs perspective on the role of each healthcare team in the care-pathway, mainly its access and support provided, and how articulation and communication among teams occur.

#### 3.4.1. Hospital Care Team Access and Support

Ongoing support is mainly provided through regular appointments with the pulmonologist but depending on the center, appointments with the nurse or clinical physiologist can also take place. After 1 week to 1 month of the implementation of the therapy, patients are assessed and then follow up each 6 months or 1 year, depending on patients’ literacy and stability, underlying disease and its stability, therapy prescribed, etc. When patients are well adapted to HRT and in a stable condition, appointments can become more spaced in time, e.g., an appointment every year. Access to the health care team outside regular appointments varies widely depending on the center organization. In some centers the team is available to clarify doubts by telephone or through unscheduled visits to the respiratory lab, other centers do not provide this support and in case of need the only option is to anticipate an appointment. There is in general lack of support from other HCPs in the patients’ long-term follow up. Concerns regarding restricted human and material resources were raised.

*“(…) all patients that I followed up have direct access and have our phone number (…)”*

(Male pulmonologist, 65 y)

*“(…) we have the door of the physiopathology lab always open and patients may show up whenever they need”*

(Female, pulmonologist, 53 y)

*“It is always variable; we adjust based on patients’ evolution, on the occurrences that they have.”*

(Female, pulmonologist, 41 y)

*“(…) we can space in time appointments, patients will have to at least one or two consultations per year”*

(Female, pulmonologist, 41 y)

*“We try, but we are not available 100% of time, but at least we try to solve patient’s problems during the appointments”*

(Female, pulmonologist, 53%)

*“we don’t have the support from other HCPs, from nurses, from physiotherapists (…)”*

(Female, pulmonologist, 39 y)

*“we needed to have the triple of the space [pulmonology department] (…)”*

(Female, pulmonologist, 53 y)

Patients are evaluated using different parameters assessed through self-reported questionnaires, blood tests, field walking tests, electronic health records (number of exacerbations, number of emergency department visits) and through information provided by the home care team (that provide a relevant input regarding adherence and efficacy of the HRT). Suggestions to improve patients monitoring via phone and telemonitoring strategies were raised.

*“this monitoring is related with the objective verification of adherence and efficacy, this is provided by the device readings and observations provided by the home care team”*

(Male, pulmonologist, 63 y)

*“(…) maybe the ideal is not that severe patients need to go to the hospital, but instead the hospital should go to their home”*

(Male, pulmonologist, 63 y)

#### 3.4.2. Home Care Team Access and Support

After the implementation week, patients have in general a higher number of visits (and calls) in the first year of adaptation to promote the adherence and enhance the support. Then, visits start to be spaced out and the frequency differ considering the type of HRT (e.g., oxygen or ventilation). However, a permanent follow-up is always maintained through different communication channels. The HCPs highlighted that the frequency of these contacts are personalized based on patients’ status, literacy, type of therapy used and adherence. At these visits, the home care team reviews the suitability of the interfaces, carries out a technical check of the equipment, reinforces the therapy education and clarifies any existing issues and perform the monitoring of adherence, which is stated to be easier to assess for HMV than LTOT. In addition to these scheduled follow up visits, home care team is accessible 24 h a day through phone contacts and extra home visits can take place. Additionally, the home care services are sometimes available in the form of open-door clinics for patients’ support.

*“they [homecare providers] are available 24 h a day”*

(Male, pulmonologist, 63 y)

*“we are lucky to have the homecare providers for HRT patients’ support”*

(Female, pulmonologist, 53 y)

*“when the patient uses a device for 16 h a day, and is stable, he has a visit every month”*

(Female, clinical physiologist, 29 y)

#### 3.4.3. Primary Health Care Team

In the current model, the role of the general practitioner is linked mainly to HRT prescription renewals. In the HCPs perspective this is an unnecessary step that produces additional burden for patients and general practitioners, linked mainly to costs assignments inside the national health service. Suggestions to improve the performance of the electronic prescription tool and also the awareness of our decision makers about the incapacitating nature of a respiratory disease emerged in HCPs reports.

*“(…) due to costs assignments to regional health administrations or to hospitals (…) the first prescription is made by the pulmonologist (…) and from then on the patient renews at his primary care centre. This constitutes a burden to the patient”*

(Male, pulmonologist, 65 y)

*“we are forcing patients in a fragile situation to go to a general practitioner, that most of the times do not know the patient and its condition, some patients do not have a general practitioner and need to spend hours waiting in their primary care centers (…)”*

(Male, pulmonologist, 65 y)

*“sometimes it is almost impossible to be able to make a renovation and that (…) moved away some general practitioners. Spend 2 h with the system and not be able to do anything, obviously is very frustrating”*

(Male, pulmonologist, 39 y)

*“(…) our political and health authorities, do not treat a chronic respiratory patient, that needs to carry oxygen, the same way as a person with motor disability, for example, a respiratory patient is not entitled to a parking permit (…)”*

(Male, pulmonologist, 63 y)

#### 3.4.4. Articulation between Health Care Teams

Articulation occurs mainly between hospital and homecare teams, which is generally good but there is still room for improvement. The hospital care team considers the articulation with the home care team essential, namely the feedback and alerts provided, although recognizing that this communication is not standardized, occurring through phone, e-mail, or paper reports. The general perception of the hospital team is that home care teams are very well prepared and commonly provide more services than the contracted. Home care team, in turn, recognizes that information received through the prescription form is short and not enough to provide adequate care, so additional contacts between the hospital and homecare team are commonly need.

*“the HCPs of the homecare providers are not supposed to be present in our appointments, but if we need them, they are there”*

(Female, pulmonologist, 53 y)

*“there is a lot of heterogeneity in the way how homecare providers contact with the physicians (…)”*

(Male, pulmonologist, 39 y)

*“(…) when ventilation is prescribed to a patient, the homecare provider receives only the prescription form, nothing more. But the prescription form does not include any information on what was assessed at the hospital, neither additional information provided by the physician (…). An email with this information would be useful”*

(Female, physiotherapist, 29 y)

*“(…) the articulation with other specialties is fundamental and articulation with the homecare providers needs to be improved (…)”*

(Male, pulmonologist, 63 y)

## 4. Discussion

Our study aimed to explore HCP’s perspective about the organization of the model of home respiratory care. HCPs have in general a very positive view, which was revealed in four major topics: Prescription, Implementation and maintenance, Carer involvement, and Quality of healthcare. The analysis of these different topics allowed us to identify specific points where improvements are needed, and which may contribute to a continuous improvement model [19].

Access and support provided from hospital care teams was distinct across centers, with some being easily available both presential or by phone outside scheduled appointments, and others not. Access issues were also raised by patients and carers [9], which is not surprising considering the strain imposed by the growing number of patients needing HRT to hospital services. Access could be improved through scheduled follow-up phone/videoconference appointments and telemonitoring strategies, as suggested by HCPs. Share experiences among centers could be also helpful. In addition, support in some centers was grounded in pulmonologist appointments, while others also provided appointments with nurses and clinical physiologists to reinforce education and support adaptation to therapy. The support of other HCPs from hospital care teams was highlighted by patients [20,21]. In a recent study about the organization of home mechanical ventilation in Portugal, the heterogeneity of the resources, both technical and human, across centers was also marked [6]. It is crucial that organization of centers evolve around multidisciplinary work to meet the needs of patients and carers [22]. In addition, the creation of a national registry for HRT is required to identify the best practices and to standardize disease management processes [6]. This registry, similarly, to other international examples [23,24], would have the potential to improve patients’ management and inform evidence-based healthcare policies.

Education of patients at the time of the initial prescription of HRT is a good example of the absence of a multidisciplinary and integrative approach. In most cases information about the need of HRT is the responsibility of the pulmonologist (or other medical specialists) during appointment, that recognize that content provided depends on the patients’ literacy and commonly do not include discussion of possible adverse events [9]. It was interesting to notice that pulmonologists considered there was space for doubts during consultations, while patients had an opposite experience [9]. The importance of adequate time to ask questions was also raised by patients from other countries, especially at the point of diagnosis [21], as the specialist physician and centre are considered their primary source of information. Written information is restricted to prescription forms, which only includes short information regarding the protocol to be implemented and to the therapy manual (that includes information about the therapy, contacts, safety, equipment guidance and cleaning). However, this information is different between each homecare provider. The information that patients receive and remember is critical for their future adherence to treatment [25] and their safety, and thus improving efficiency in education is critical. This is in line with Portuguese patients’ perspective [9]. This need has also been highlighted patients on home oxygen therapy from Spain [20] and by patients with idiopathic pulmonary fibrosis from 4 European countries [21]. The involvement of carers was not unanimous, with some HCPs considering the involvement of the carer dependent on the patient’s autonomy. This was mainly attributed to carers’ excessive support and protection. Yet, from the patient perspective, carers’ support is essential [9]. Patients want their partner attending their medical appointments and being involved in the care pathway [21]. Educating and supporting carers should also be targeted, which can potentially normalize negative behaviors, such as total denial or overprotection [21]. The responsibility for patients’ and carers’ education needs to be shared with other HCPs beyond the hospital and home healthcare setting. Crucial written information should be provided in plain language [21,26,27], easily irrespective of the literacy level and should be standardized among hospital, primary care and homecare understandable teams. This shared responsibility and reinforcement of education at distinct moments has potential to improve patients’ acceptance of the HRT and long-term adherence [28].

Issues with HRT prescription were raised, namely related with usability of the medical electronic prescription system (PEM-CRD), its restriction to the public health sector (that excludes private sector) and, mainly, the burden of prescriptions renewal. Since its development and implementation, the medical electronic prescription system has been subject to successive improvements [29], nevertheless a usability study is required to identify critical points still to be enhanced. This would probably impact on physicians’ experience with the system, namely the general practitioners. The restriction to public sector is also a problem, as it delays prescription of HRT to patients in need and increases the pressure on healthcare services. Renewal of prescriptions is done according to the therapy (different therapies have different renewal timings and settings), and not according to the patient. This can lead to a situation where a patient receiving three therapies needs to renew them on different dates and settings (hospital and primary care) [8]. This pathway is clearly not aligned with a patient-centered care model and a serious reflection about its need is urgent. This difficulty was also reported previously by Portuguese patients, which suggested that renewal process should be target of reflection and reassessment [9]. This concern was not raised by patients in other studies [20,21]. This is probably related to the fact that in some countries home respiratory therapies are considered lifelong needs for chronic patients and thus therapy renewals are not required.

According to the Portuguese model of home respiratory care, there is a national geographical equity on the access to all therapies and patients are free to decide which homecare provider will deliver the respiratory therapy. In the HCPs’ perspective this is a strong point of the current model, as it creates competitiveness among the homecare providers homologated for the Portuguese Ministry of Health to delivering HRT in Portugal, which in their view raises the quality of the services provided. The general perception of the hospital care teams is that home care teams have a performance above what is contracted. This links to the good perception both patients and carers have of the support received from the home care teams [9]. The reality in other countries, such as Spain, is distinct, with patients having contradictory positions depending on the geographical area [20]. At present, the choice of the homecare provider is most commonly a “guided choice” as mentioned by HCPs, as patients do not have access to any specific information, besides the contacts of the different companies. To improve patient’s autonomy and informed decisions, easily readable information about quality indicators of each homecare provider need to be available. This will only be possible when a core set of key performance indicators (KPI) are selected and start to be routinely assessed. Most importantly, these KPI can be nationally used to evaluate the quality of HRT provided and should include not only Patient-reported outcome measures (PROMs) but also patient patient-reported experience measures (PREMS). Moreover, can become the basis of a value-based health care model (VBHC).

In the HCPs’ and patients’ views, the role of the general practitioner is linked mainly to HRT prescription renewals. This is not surprising as articulation occurs mainly between hospital and homecare teams, with primary care team not being kept up to date about patient care. Patients with idiopathic pulmonary fibrosis from 4 European countries also wanted specialist physicians always to communicate their diagnosis to their local doctor with details about their treatment and reported lacking confidence in local doctors who were perceived to have less depth understanding of the disease [21]. Communication among the healthcare teams should be improved and primary care teams specifically trained about home respiratory therapies.

This study has some limitations that need to be acknowledge. HCPs involved in the management of patients with chronic respiratory failure receiving HRT, but with different clinical backgrounds, working experience and from distinct settings and regions were included to build a comprehensive perspective regarding the national home respiratory care organization. Nevertheless, we need to have caution interpreting this work as it does not address sleep care and it may be biased to pulmonologists’ view since they were the larger professional group. In future, sleep care could be also included, and a balance sample should be targeted. Moreover, HCPs from primary care were underrepresented (with only one nurse participating) and the 5 HCPs integrating homecare teams (4 clinical physiologists and 1 physiotherapist) were workers of the same homecare provider. It would thus be relevant in future studies to integrate the perspective of general practitioners as well as from HCPs from other homecare providers delivering HRT in Portugal. The agreement between the two researchers has been performed for one random focus group. But we are confident regarding the rigor of the results presented as agreement was found to be high and the preliminary results were validated by 5 healthcare professionals. We recognize that HCPs perspective is only one part of a broader picture, that needs to be combined with the perspective of other stakeholders, such as patients, carers, researchers and decision-makers to improve health services. We recently explored the experience of patients and carers [9]. Also, Clèries et al. showed how patients have different views on service delivery. Patient complaints, grievances, or comments are a very important source for introducing improvements [20]. Nonetheless, studies addressing priorities and information needs of homecare providers and decision makers are still lacking. Only by integrating the perspective of the different stakeholders involved the current HRT model, it will be possible to identify the major drawbacks and aspire for an improved and integrated healthcare model. The careful and kind care model could be a good reference and, as Allwood et al. suggested, healthcare should be “elegant” (no waste or haste), “focused” on the relevant elements of biology and biography, “sensitive” to each patient’s problems and “minimally disruptive”, with little interference in the lives of patients [30].

## 5. Conclusions

HCPs have a very positive view about the Portuguese model of home respiratory care, but their perspective allowed us to identify specific points where improvements and reflections are needed that are mainly related to patients’ late referral, renewals burden HRT prescription and lack of articulation between hospital, primary and home care teams. This knowledge may be useful to decision makers improve the current healthcare model.

Future studies should explore the perspective of homecare providers, researchers, and decision makers to contribute for an improved and integrated HRT model.

## Figures and Tables

**Table 1:** Characteristics of healthcare professionals (*n* = 28).

Characteristic	Number	Title
Female, *n* (%)	19	(68)
Age, median [p 25–p 75] y	42	[37–53]
Working experience, median [p 25–p 75] y	19	[13–28]
Region, *n* (%)		
Porto	12	(43)
Lisboa	10	(36)
Coimbra	6	(21)
Background, *n* (%)		
Pulmonologist	16	(57)
Clinical physiologist	8	(29)
Physiotherapist	2	(7)
Nurse	2	(7)
Sector, *n* (%)		
Public	10	(36)
Private	5	(18)
Both	13	(47)
Setting, *n* (%)		
Secondary care	22	(79)
Home care	5	(18)
Primary care	1	(4)

p 25–p 75, percentile 25–percentile 75.

**Table 2:** Frequency in units of meaning of each identified topic and sub-topic during focus groups.

Topics	Sub-Topics	Units of Meaning	Frequency
Prescription			171
	Setting and HCP roles	Referral Context HCP Roles Administrative issues	92
	Education	Type and goals of the therapy Benefits of the therapy Home care provider choice Exacerbations action plan Space for doubts Written information Role of patients’ associations	88
Implementation and maintenance			162
	Setting and HCP roles	Context HCP Roles Adaptation to therapy Concerns about multidisciplinary approach	71
	Education	Benefits of the therapy Space for doubts Information about the equipment Safety information Home care team access and support Written information	38
	Quality of life impact	Social impact Emotional impact Professional impact Impact on activities of daily living	37
	Adherence	Patient profiles Experienced benefits	43
Carer involvement	-	Physical support Emotional support Communication with the healthcare team	65
Quality of healthcare			247
	Hospital care team	Follow up based on regular appointments Difficult access outside appointments Concerns about multidisciplinary approach Patients’ monitoring	107
	Home care team	Follow up through home visits and phone contacts Easy access outside planned contacts Therapy adherence monitoring	78
	Primary care team	HRT prescription renewals Administrative issues	21
	Articulation between healthcare teams	Hospital-home teams Communication issues	70

## Data Availability

The data presented in this study are available on request from the corresponding author.

## Appendix A. Focus Groups Semi-Structured Discussion Guide

How HRT are prescribed? With facilitators/barriers you encounter?

How and who made the decisions about the treatment?

Are different healthcare professionals involved? What are their roles?

What is the role of the patient and of the carer?

What information is provided at this stage? And how is provided?

How treatment begins?

Who is involved?

How training of patients and carers occur?

What information is given at this point? And how is provided?

What is more important at this point? Which are the main difficulties?

How patients are follow-up?

How integration of the different healthcare professionals occurs?

How do you assess the treatment results? Which measures/indicators are used?

What could be improved?

Which are the critical points (advantages and disadvantages) of the current respiratory care model?

What could be done to improve the model?

Have we addressed all the relevant topics relevant for you? Do you would like to add anything?
